# High Output Performance and Ultra-Durable DC Output for Triboelectric Nanogenerator Inspired by Primary Cell

**DOI:** 10.1007/s40820-022-00898-2

**Published:** 2022-08-02

**Authors:** Shaoke Fu, Wencong He, Huiyuan Wu, Chuncai Shan, Yan Du, Gui Li, Ping Wang, Hengyu Guo, Jie Chen, Chenguo Hu

**Affiliations:** 1 Department of Applied Physics, State Key Laboratory of Power Transmission Equipment and System Security and New Technology, Chongqing University, Chongqing, 400044 People’s Republic of China; 2 College of Physics and Electronic Engineering, Chongqing Normal University, Chongqing, 401331 People’s Republic of China

**Keywords:** Triboelectric nanogenerator, DC output, Durability, Intelligent monitoring

## Abstract

Triboelectric nanogenerator (TENG) is regarded as an effective strategy to convert environment mechanical energy into electricity to meet the distributed energy demand of large number of sensors in the Internet of Things (IoTs). Although TENG based on the coupling of triboelectrification and air-breakdown achieves a large direct current (DC) output, material abrasion is a bottleneck for its applications. Here, inspired by primary cell and its DC signal output characteristics, we propose a novel primary cell structure TENG (PC-TENG) based on contact electrification and electrostatic induction, which has multiple working modes, including contact separation mode, freestanding mode and rotation mode. The PC-TENG produces DC output and operates at low surface contact force. It has an ideal effective charge density (1.02 mC m^−2^). Meanwhile, the PC-TENG shows a superior durability with 99% initial output after 100,000 operating cycles. Due to its excellent output performance and durability, a variety of commercial electronic devices are powered by PC-TENG via harvesting wind energy. This work offers a facile and ideal scheme for enhancing the electrical output performance of DC-TENG at low surface contact force and shows a great potential for the energy harvesting applications in IoTs.

**Supplementary Information:**

The online version contains supplementary material available at 10.1007/s40820-022-00898-2.

## Highlights

- A novel structure of the PC-TENG based on triboelectrification and electrostatic induction is designed.
- The PC-TENG produce ideal direct current output for driving small electronic devices directly even at low surface contact force.
- The novel PC-TENG owns a high durability and high output performance due to low wear of materials.
- The R-PC-TENG can efficiently harvest ambient mechanical energy.

**Supplementary Information:**

The online version contains supplementary material available at 10.1007/s40820-022-00898-2.

## Graphical abstract

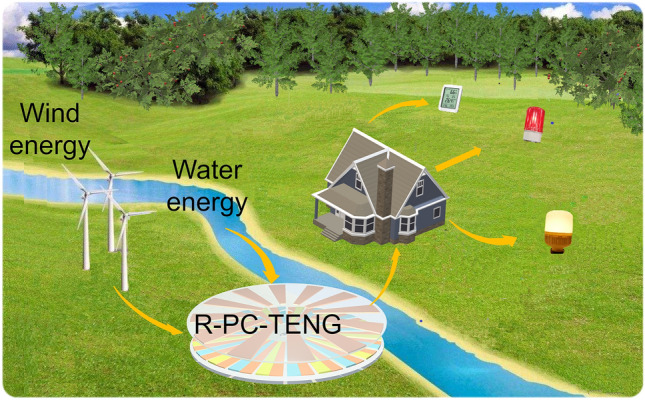

## Introduction

With rapid growing of Internet of Things (IoTs) and Big Data (BD) in the new era, billions of distributed sensor networks have been developed to collect and transform information from ambient environments [1–4]. These distributed sensors drive urgent demand and bring huge challenge to distributed and sustainable power supply [5–8]. Fortunately, triboelectric nanogenerator (TENG), as a new energy converter, provides a promising effective technique for scavenging low frequency mechanical energy, such as vibration energy [9–11], wind energy [12–14], water flow energy [15, 16] and converting into electric energy. Besides, TENG shows great merits in large scale applications for IOTs, BD, and environmental monitoring, such as cost-effectiveness, abundant materials availability, simple structure design, and environmental friendliness [17–21].

Various TENGs, according to their working mechanism and output signal type, have been roughly divided into two categories: (I) Based on the coupling of triboelectrification and electrostatic induction, the TENG generates an alternating current (AC-TENG) [22–26]; (II) Based on the coupling of triboelectrification and air-breakdown, the TENG produces a direct current (DC-TENG) [27–30]. Among them, the DC-TENG can supply power directly to electronic devices without rectifier circuits and energy storage units. At present, the effective charge density of the DC-TENG based on air breakdown has reached overall output charge density by integrating DC-TENG units [30]. However, the DC-TENG lacks the charge accumulation process during operation, its effective charge density depends heavily on the surface contact force. It is obvious that strong mechanical wear occurs due to high surface contact force, which is a bottleneck problem for TENG’s commercial applications. It is interesting to note that the AC-TENG based on triboelectrification and electrostatic induction can continuously accumulate triboelectric charge. AC-TENG can produce ideal effective charge density even at low surface contact force. However, as most electronic devices need DC power, AC-TENG cannot directly drive them without rectifier circuits. It is worth noting that the remarkable characteristic of the primary cell is a constant DC output, and its basic working principle is the metal activity difference between the two metal electrodes in the electrolyte [31–33]. Similarly, the basic principle of triboelectrification is based on differences in the electronic affinity of different materials. Therefore, it is necessary to explore a new kind of the DC-TENG based on the basic structure of primary cell [34–36].

To achieve high durability and high output performance DC-TENG, herein, inspired by primary cell and its DC output characteristics, a novel primary cell structure TENG (PC-TENG) is proposed, which can continuously accumulate triboelectric charge like AC-TENG, and can produce ideal DC output for directly driving small electronic devices at low surface contact force of 12 N. The carbon gel and appropriate thickness of foam was used to optimize the contact state between the friction layers to further reduce the material wear and prepare the stepped structure, respectively. For demonstration, a vertical contact-separation mode CS-PC-TENG and a free-standing mode FS-PC-TENG are fabricated and the theoretical mechanism and influence factors of the charge transfer in each working cycle are systematically analysed. Meanwhile, a rotary R-PC-TENG is designed with a diameter of 180 mm and its maximum effective charge density reaches 1.02 mC m^−2^. The PC-TENG shows a superior durability with 99% initial output after 100,000 operating cycles. Besides, the R-PC-TENG is used to charge various commercial capacitors quickly and power 944 green light-emitting diodes (LEDs) to high brightness in series. Furthermore, the R-PC-TENG is also used to power a commercial infrared transmitter module and four thermo-hygrometers in parallel by harvesting wind energy. This work provides a facile and ideal scheme for enhancing the electrical output performance of DC-TENG at low surface contact force and shows a great potential for harvesting ambient energy for IoTs.

## Experimental

### Fabrication of the CS-PC-TENG

**Fig. 1:**
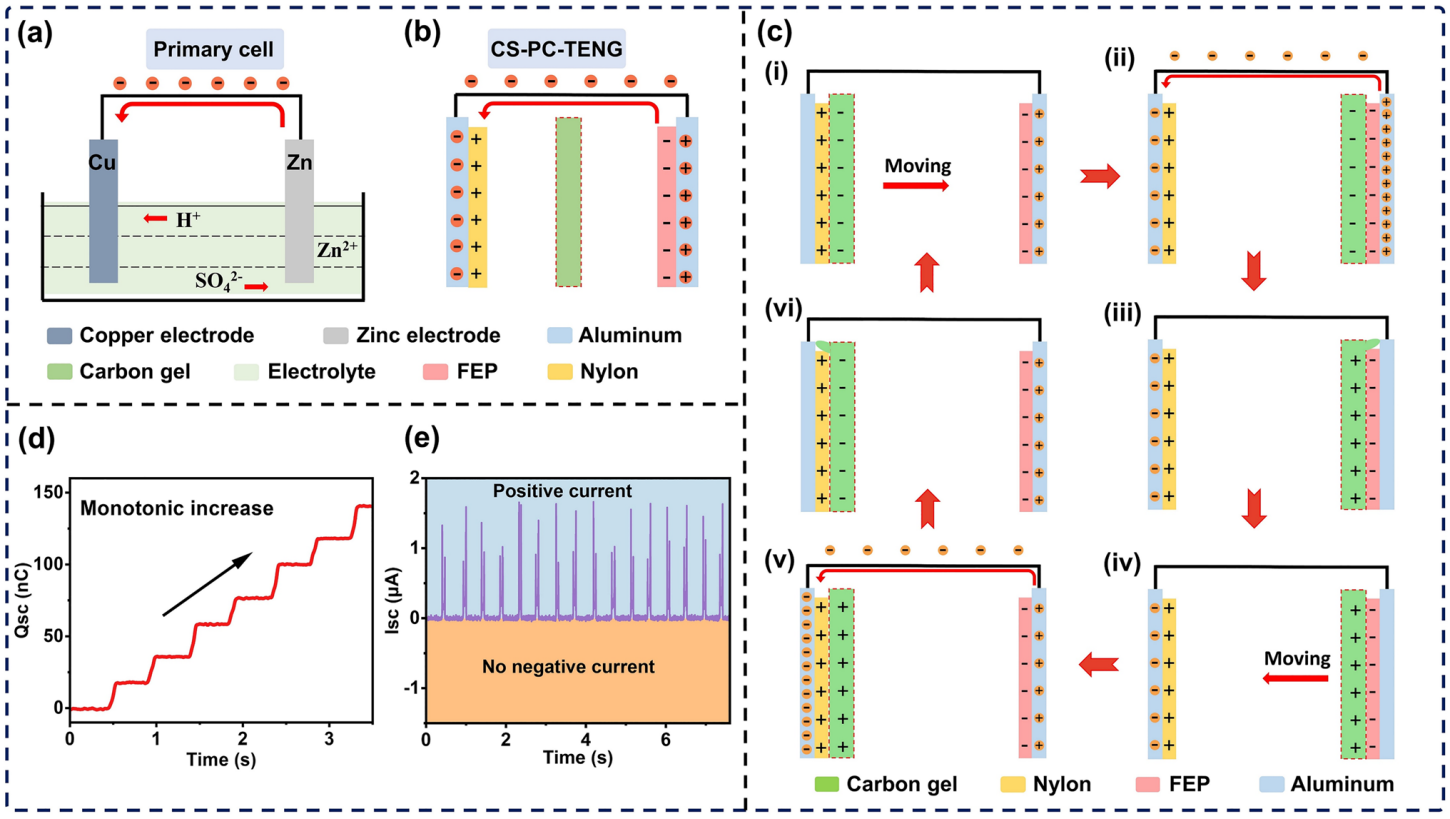
The structural comparison of primary cell and the CS-PC-TENG, and working mechanism of the CS-PC-TENG. **a** Basic structure of the Zn/Cu primary cell. **b** Basic structure of the CS-PC-TENG. **c** Schematic diagrams of the working principle of the CS-PC-TENG. **d, e** The basic output charge and current of the CS-PC-TENG

As shown in Fig. 1b, the CS-PC-TENG consists of three parts, a negative friction electrode, a positive friction electrode, and a conductive carbon gel, which correspond to the cathode, anode, and electrolyte of the primary cell, respectively. For the negative friction electrode, its preparation process is as follows. First, 20 mm × 25 mm × 25 μm aluminium foil was pasted on a 5 mm thick acrylic substrate, which was cut by a laser cutter to the dimensions of 40 mm × 40 mm × 5 mm. Then, 20 mm × 20 mm × 25 μm FEP film was stuck to the upper surface of the aluminium foil. For the positive friction electrode, the preparation process is the same as that for the negative friction electrode, except that the 25 μm FEP film was replaced by a 25 μm nylon (PA) film. For the conductive carbon gel, an amount of Parts A of Ecoflex 20 was placed in a petri dish. Then, an appropriate proportion (10% of the total mass) of carbon powder to Parts A of Ecoflex 20 was dispensed and stirred for 10 min to get a mixed liquid. Parts B of Ecoflex 20 equal to Parts A was added to the mixed liquid. After fast stirring for 10 min, the mixed liquid was transferred into the square acrylic groove, and squeezed for 24 h with another acrylic substrate. Then the preparation of the conductive carbon gel was completed.

### Fabrication of the FS-PC-TENG

The FS-PC-TENG consists of a stator and a slider. For the slider, the copper foil with thickness of 50 μm was attached to the 50 mm × 15 mm × 5 mm acrylic substrate, and 10 mm × 5 mm × 25 μm spring steel sheet was pasted on the edge of the substrate as a brush. The brush was connected to the copper foil. For the stator, a 5 mm thick acrylic plate was cut into an appropriate size as the substrate. The foam of appropriate thickness was cut to the same area as the slider and was affixed to the surface of the substrate. Three aluminium electrodes with a gap of 3 mm were pasted on the foam. Connecting the first and third electrodes together with a wire as output terminal 1, and the second electrode as output terminal 2. Besides, PA and FEP film were alternately pasted on the electrodes as friction layers. Finally, a pin was extended at the edge of each electrode, and when the slider was directly above the friction layer, the brush could contact the pin.

### Fabrication of the R-PC-TENG

The R-PC-TENG contains a stator and a rotator. The preparation process is similar to that of the FS-PC-TENG. For the rotator, the acrylic plate with thickness of 5 mm was cut into a disc with an inner diameter of 60 mm and an outer diameter of 160 mm. The copper foil with thickness of 50 μm was attached to acrylic disc and then cut into 12 equally shaped sectors to serve as friction electrodes. Then, the edge of each electrode was connected to a brush, which was made of spring steel with dimension of 10 mm × 5 mm × 25 μm. For the stator, a 5 mm thick acrylic plate was cut into a disc as a substrate with radial stripe, and the inner and outer diameter of the disk is 60 and 180 mm, respectively. The fan-shaped foam was attached to the substrate and its area is equal to that of the copper foil of the stator. A 25 μm aluminium foil was attached to the acrylic disc and cut into 12 pairs of scalloped electrodes with same shape as the output electrode pairs. The PA and FEP film were alternately pasted on the electrodes as friction layers. Finally, a pin was extended at the edge of each electrode, and when the slider was directly above the friction layer, the brush could contact the pin.

### Electrical Output Measurement and Characterization

The plane-type FS-PC-TENG slider was driven by a linear motor (LinMot S01-72/500). A programmable stepper motor (86HSE8.5 N) is used to drive the rotation of rotation-type R-PC-TENG. The short-circuit current and the transferred charge of the TENG were measured by a Keithley 6514 Electrometer and the open-circuit voltage was tested by a high-speed electrostatic voltmeter (Trek model 370). In application demonstrations, a wind power generation system with adjustable wind speed was established to simulate the natural wind. A 750 W low-power water pump is used to generate the water flow to simulate the water flowing down the mountain.

## Results and Discussion

### Structure Design and Working Principle of the CS-PC-TENG

The TENG based on triboelectrification and air-breakdown produces a direct current signal. However, high output performance of this kind of TENG depends largely on strong surface contact force and has a poor durability. To achieve high output performance for a TENG with direct current output at low surface contact force, herein, inspired by primary cell and its DC signal output characteristics, a novel PC-TENG is proposed. Figure 1a shows the basic structure of the Zn/Cu primary cell. Because of the difference in metal activity, the zinc electrode (negative electrode) loses electrons and forms zinc ions in the electrolyte, and then, hydrogen ions gain electrons at the copper electrode (positive electrode) to form hydrogen gas. In this process, hydrogen ions directly flow from anode to cathode in the internal electrolyte. At the same time, electrons directly flow from negative electrode to positive electrode along the external circuit and form a DC output signal. In order to mimic the metal activity differences of primary cell, a PC-TENG is prepared by constructing three tribo-materials with different electronegativity. The structure and working principle of PC-TENG are described in detail below. Figure 1b exhibits the basic structure of the contact-separation PC-TENG (CS-PC-TENG), which has a similar structure to a primary cell. The left electrode attached to the back of the nylon (PA) film acts as the positive electrode (copper electrode in PC), and the right electrode attached to the back of the FEP film serves as the negative electrode (zinc electrode in PC). The carbon gel functions as electrolyte in PC, forming positive charge (hydrogen ions) and negative charge (sulfate ion) movement in a directional way in the PC-PENG, respectively. The detailed working principle of DC output is shown in Fig. 1c, where the electron affinity of the material is FEP > carbon gel > PA in the triboelectric series [37]. Therefore, in stage i, the PA is positively charged on contact with carbon gel, while carbon gel is negatively charged. In stage ii, the carbon gel with negative charge moves from left to right and contacts with the FEP film and causes a strong potential difference between the dielectric materials. In this process, the charge transfers from right electrode (back of the FEP film) to left electrode (back of the PA film) along the external circuit due to electrostatic induction. In stage iii, the carbon gel continuously moves to the right and contacts the right electrode due to its soft deformation (on the top). Therefore, partial charge is neutralized and the carbon gel has positive charge. In stage iv, the carbon gel starts to move in the opposite direction and separates from the right electrode. In stage v, the carbon gel with positive charge moves from right to left and contacts with the PA film. In this process, a strong potential difference is created between the dielectric materials, and the charge transfers from the right electrode to the left electrode through the external circuit again. In stage vi, the carbon gel continuously moves to left and contacts with the left electrode. At this moment, partial charge is neutralized and the carbon gel has negative charge because carbon gel is in close contact with the PA film. Last, the carbon gel starts to move in the opposite direction and separates itself from the left electrode, which completes one cycle. As the carbon gel moves right and left periodically, electrons flow from the right electrode to left electrode. Besides, the carbon gel is positively charged as it moves from right to left, which is similar with the hydrogen ion in the primary cell. Then, the carbon gel is negatively charged as it moves from left to right, which is similar with the sulfate ion in the primary cell. The structure of the CS-PC-TENG is similar with that of the primary cell, and it has the same DC output characteristics as a primary cell. As shown in Fig. 1d–e, both the transferred charge and the short-circuit current show DC output feature.

### Influence of the Material and Structure

**Fig. 2:**
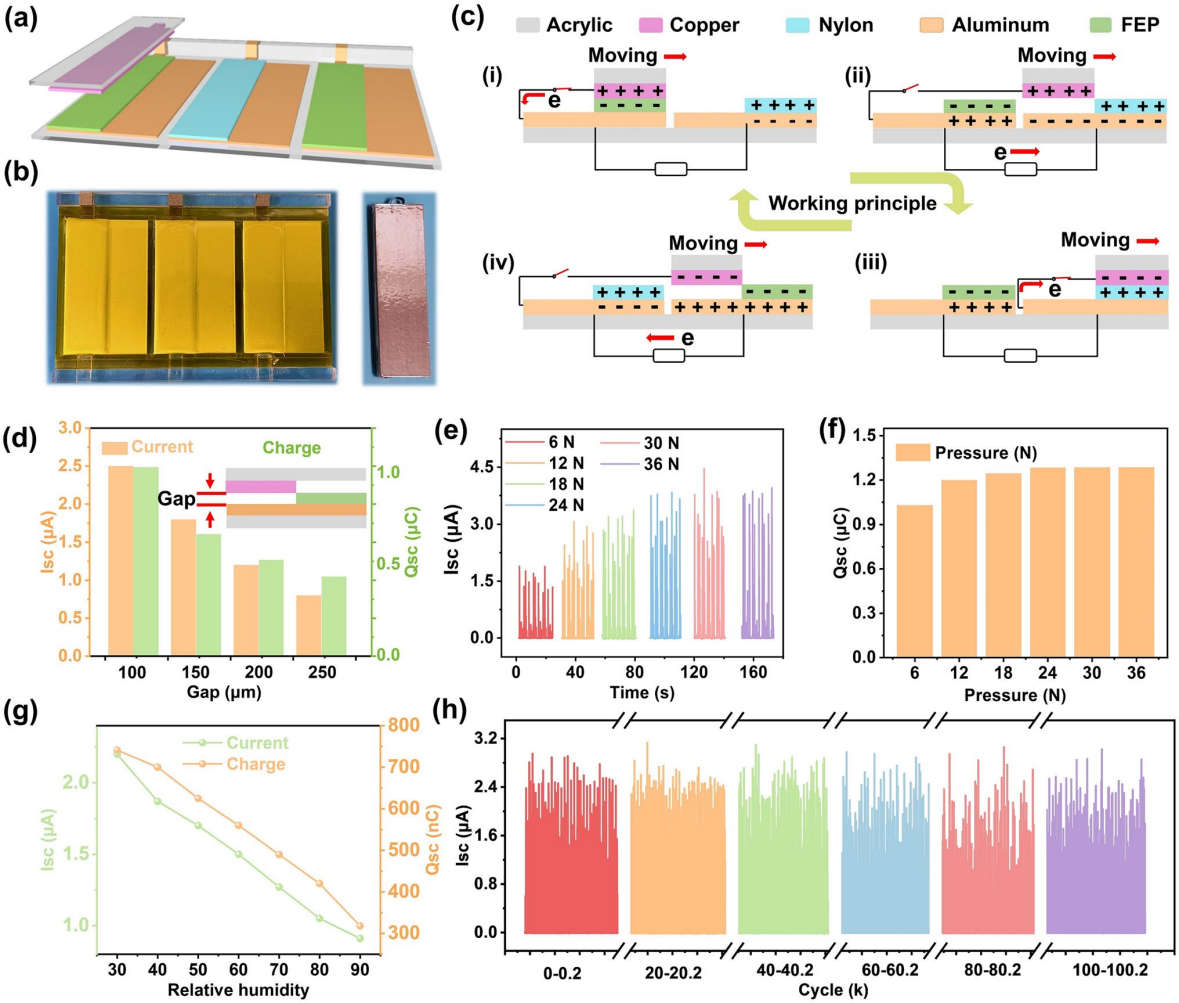
The structure, working mechanism, and influence factors on the output performance of FS-PC-TENG. **a, b** 3D structural scheme and photographs of the FS-PC-TENG. **c** Schematic diagrams of working principle of the device. The basic short-circuit current and charge output of the FS-PC-TENG at **d** different air gaps, **e, f** different contact force, **g** different humidity. **h** The stability and durability of the FS-PC-TENG

Generally, four different working modes of TENG can be reasonably designed according to different application scenarios, such as contact-separation mode (CS), single electrode mode, plane sliding mode, and freestanding mode (FS), among which FS-TENG can efficiently convert mechanical energy and is the best way to realize rotational motion through gas/liquid fluid driving force [38–41]. Therefore, the PC-TENG is designed in FS mode denoted as FS-PC-TENG, which working principle is same as that of the CS-PC-TENG. The schematic diagram of its working principle is shown in Fig. 2a. Different electropositive materials are explored and the short-circuit current (*I*_sc_) and transferred charge (*Q*_sc_) of the FS-PC-TENG are shown in Fig. S1a-b. The FS-PC-TENG with FEP film has highest output performance. Here, the FEP and PA film with 25 μm thickness are pasted alternately on the acrylic substrate as positive and negative tribo-materials. Besides, different slider materials are explored, as shown in Fig. S2, the maximum value of FS-PC-TENG output is obtained when the slider material is copper foil. Therefore, copper foil is stuck on an acrylic slider. An appropriate thickness of foam is used to optimize the contact state between the friction layers to further reduce the material wear and prepare the stepped structure. Figure 2b shows top view photographs of the stator and slider of the FS-PC-TENG, respectively. The detailed manufacturing process for the FS-PC-TENG is in “Experimental” section. The detailed working principle for the FS-PC-TENG is presented in Fig. 2c, which is same as that of the CS-PC-TENG. Because the electron affinity of the material is FEP > copper > PA in the triboelectric series, PA film loses electrons and copper foil obtains electrons during the friction between PA film and copper foil, while the copper foil loses electrons and the FEP film gains electrons during the friction between the copper foil and FEP film. When the surface charge density is stabilized, there are four charge distribution states in a cycle of the FS-PC-TENG. In stage i, when the copper foil is in contact with the FEP film, the switch (a brush) between copper foil and aluminium electrode on the back of the FEP film is closed. The aluminium electrode on the back of the FEP film has no charge, while the electrode on the back of the PA film has negative charge. In stage ii, when the slider with positive charge moves on the blank electrode, the switch between copper foil and aluminium electrode on the back of the FEP film is open. According to electrostatic induction, electrons transfer from the aluminium electrode on the back of the FEP film to the aluminium electrode on the back of the PA film through external circuit. As the slider continuously moves, in stage iii, the copper foil is in contact with the PA film, and at the same time, the switch (a brush) between copper foil and aluminium electrode on the back of the PA film is closed. It is worth noting that the electron affinity of copper materials is greater than that of PA film, and copper is in close contact with PA film in this time. Therefore, the copper is negatively charged and the aluminium electrode on the back of the PA film has no charge, while the electrode on the back of the FEP film still has positive charges. In stage iv, when the slider with negative charges moves onto the next blank electrode, the switch between copper foil and aluminium electrode on the back of the PA film is open. Because of the electrostatic induction, the aluminium electrode on the back of the PA film generates negative charges, and the blank electrode beneath the copper foil generates positive charges. Therefore, electrons transfer from the blank electrode to the aluminium electrode on the back of the PA film through external circuit. In this process, the detailed working principle of the FS-PC-TENG is shown in Fig. S3. It is obvious that the flow direction of electrons in the external circuit is fixed during the state ii and iv. In state i and iii, although the charge on the copper foil changes, there is no charge flow in the external circuit. Therefore, periodic movement of the slider produces cyclic DC output. Because electrostatic induction and gap distance has a strong relationship, the short-circuit current (*I*_sc_) and transferred charge (*Q*_sc_) of the FS-PC-TENG at different gap distances are measured, as shown in Fig. 2d. As the gap distance increases from 100 to 250 μm, the *Q*_sc_ decreases from 1.03 to 0.44 μC and the Isc decreases from 2.5 to 0.8 μA, respectively. Figure 2e-f shows the *I*_sc_ and *Q*_sc_ of the FS-PC-TENG with different pressure force. With the pressure force increasing from 6 to 36 N, the *I*_sc_ increases from 1.75 to 3.85 μA, and the *Q*_sc_ increases from 1.03 to 1.3 μC. The *I*_sc_ and *Q*_sc_ increase rapidly and then stabilize when the pressure force is larger than 24 N, which is due to the function of support action of the gasket that lets the contact force remain constant with further increase in pressure force. To demonstrate the superiority of PC-TENG in small pressure force, the comparison of the output charge of PC-TENG with that of normal DC-TENG based on triboelectrification and air-breakdown at different pressure force are shown in Fig. S4. Obviously, although the output charge increases significantly with the increase in pressure force for DC-TENG, the output charge is much smaller than that of PC-TENG at 6 N. However, as the output charge of DC-TENG increases with the pressure force, it exceeds the output charge of PC-TENG at 36 N. It is worthy to note that the DC-TENG based on triboelectrification and air-breakdown lacks charge accumulation during operation, and its charge output depends heavily on the surface contact force, resulting in severe mechanical wear. Interestingly, the FS-PC-TENG based on triboelectrification and electrostatic induction still has an ideal output performance even at low contact force. Therefore, PC-TENG achieves high durability and high output performance at low contact force. Figure S5 shows the *I*_sc_ and *Q*_sc_ of the FS-PC-TENG with different sliding speeds. The *Q*_sc_ of FS-PC-TENG increases from 0.63 to 1.72 μC within 20 s as the sliding speed increases, and Isc increases linearly with the sliding speed from 3 to 9 cm s^−1^. These results show that the FS-PC-TENG has superior DC output characteristics due to the coupling of triboelectrification and electrostatic induction. In addition, the FS-PC-TENG demonstrates excellent durability due to the low surface contact force and soft contact effect of foam. To demonstrate good applications in the natural environment, the influence of humidity to the FS-PC-TENG is measured as shown in Fig. 2g. The *Q*_sc_ and *I*_sc_ decrease linearly with the increase in relative humidity. Besides, the stability of the FS-PC-TENG device is measured under atmospheric relative humidity. As exhibited in Fig. 2h, after 100,000 cycles, the FS-PC-TENG device displays outstanding stability and durability (maintaining 99% of initial output) owing to low surface contact force and soft contact effect of foam.

### Electrical Output Performance of R-PC-TENG

**Fig. 3:**
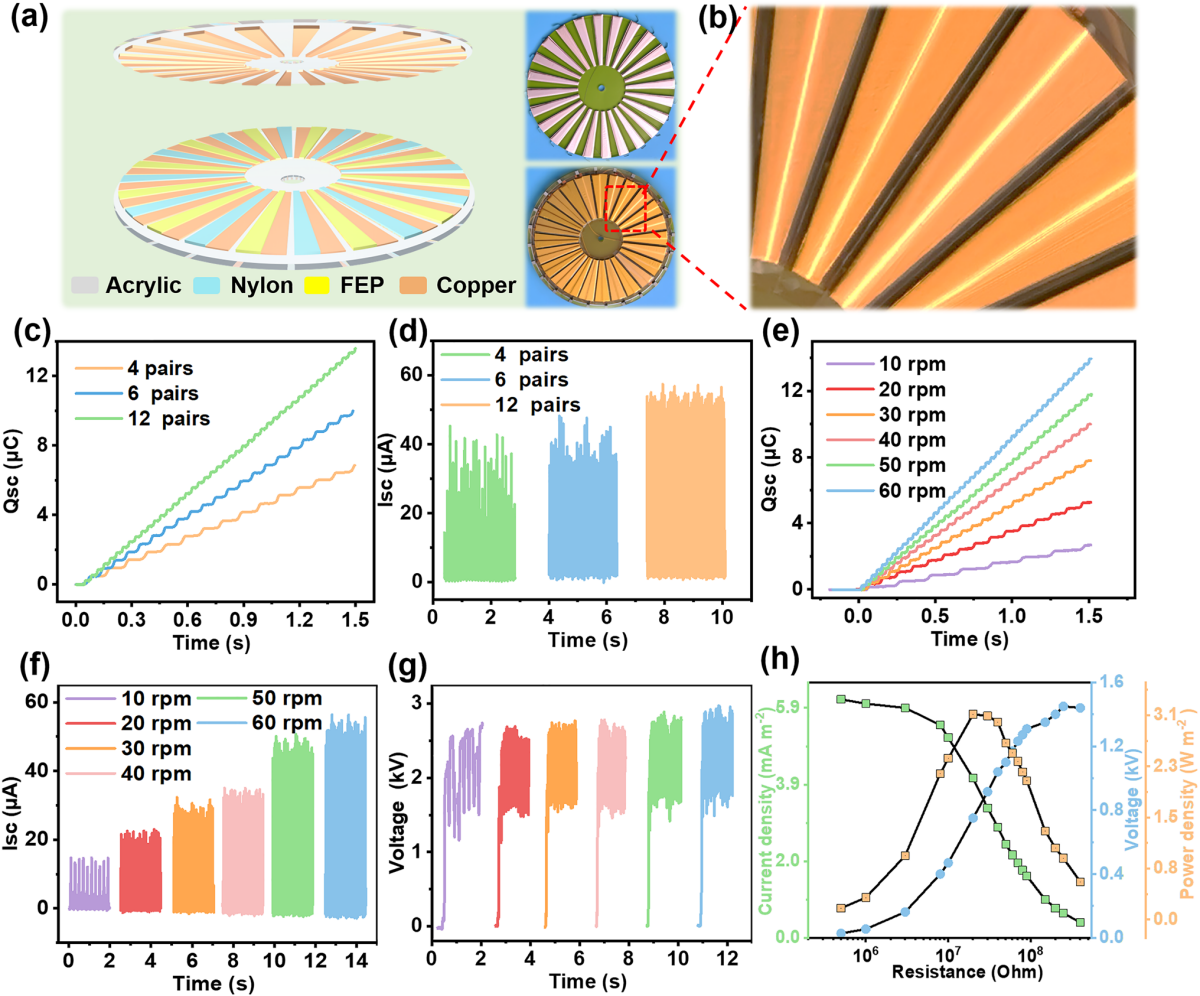
The structure and performance of the R-PC-TENG. **a** 3D structural schematic of the R-PC-TENG device, and photographs of rotator and stator parts. **b** Partial enlarged detail of stator. **c, d** Transfer charge (*Q*_sc_) and short-circuit current (*I*_sc_) of R-PC-TENG with different pair of electrodes. **e–g** Transferred charge (*Q*_sc_), short-circuit current (*I*_sc_), and open-circuit voltage of the R-PC-TENG at different rotational speeds. **h** Evaluation of matching impendence and output power of the R-PC-TENG at rotational speed of 60 rpm

In order to efficiently harvest the energy of wind and water flow in the wild/outdoor environment, based on the proposed mechanism and parameters optimization, the parallel FS-PC-TENG is changed into the radially arrayed rotary mode R-PC-TENG. 3D structural scheme and the top view photographs of the R-PC-TENG are shown in Fig. 3a, which include a stator and a rotator, respectively. Both the stator and rotator consist of several sectors, each sector unit is similar with that of the sliding mode parallel FS-PC-TENG. Figure 3b shows the enlarged view of local structure of the stator, it can be clearly seen that the radially arrayed electrode has a stepped structure. The height of the stepped structure and the size of the surface contact force are same as the parameters of FS-PC-TENG. In order to further optimize the DC output performance of R-PC-TENG, the number of the electrodes of R-PC-TENG is studied as shown in Fig. 3c–d. The *Q*_sc_ within 1.5 s and *I*_sc_ of the R-PC-TENG increase with the increase in electrode pairs, and the output charge density of the R-PC-TENG reaches 1.02 mC m^−2^ calculated according to the area of the slider at 12 pairs of electrodes. Higher output performance could be obtained through by precise machining to improve the integration of electrode pairs. Besides, to reveal the relationship between rotation speed and output performance of the R-PC-TENG, the transferred charge, short-circuit current, and open-circuit voltage are displayed in Fig. 3e–g. The transferred charge of the R-PC-TENG reaches 14 µC in 1.5 s at the rotational speed rising to 60 rpm. Besides, the short-circuit current and the open-circuit voltage are about 56 µA and 2.8 kV at 60 rpm, respectively. Figure 3h displays the short-circuit current, open-circuit voltage and peak power of the R-PC-TENG at different resistance from 0.5 to 400 MΩ, and the maximum peak power reaches 28 mW with resistance of 20 MΩ at 60 rpm. To sum up, this work achieves an ideal DC output performance for the R-PC-TENG via the coupling of triboelectrification and electrostatic induction.

### Application of R-PC-TENG for Wind Energy Harvesting

**Fig. 4:**
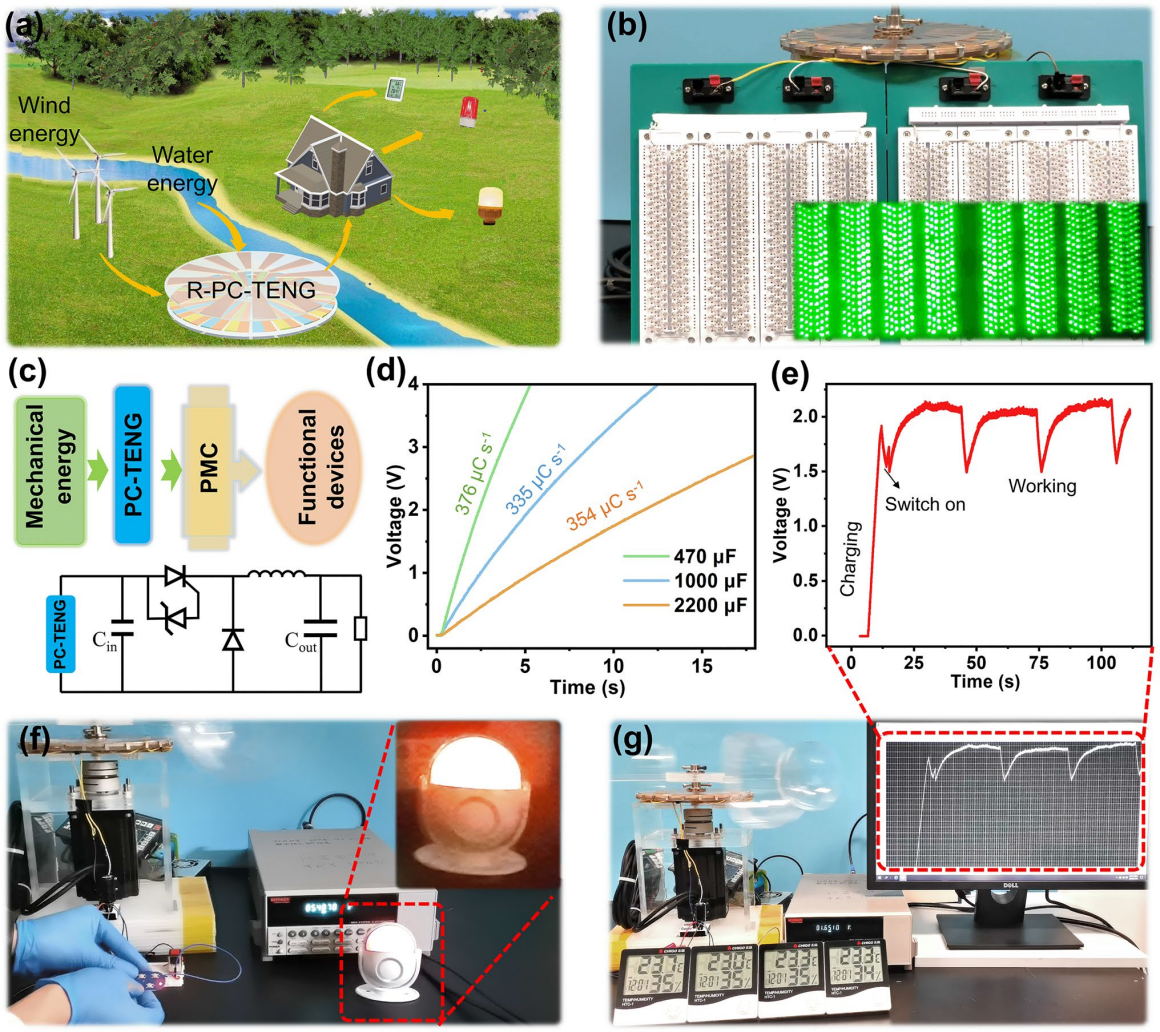
Application of the R-PC-TENG powering different electronic devices. **a** The application scenario proposed for the R-PC-TENG. **b** Demonstration of directly powering 944 LEDs at 60 rpm. **c** System diagram and circuit diagram of the self-powered system for powering electronics with power management. **d** Voltage curves of charging 470, 1000, and 2200 μF capacitors by using R-PC-TENG device at rotational speed of 60 rpm. **f** Charging 2.2 mF capacitor while powering an infrared transmitting module under the driven of wind. **e, g** Charging 470 μF capacitor while powering four hydro-thermometers by wind energy

In order to visualize practical applications of the R-PC-TENG in nature environment, the prospect of the R-PC-TENG for wind and water flow energy harvesting is illustrated in Fig. 4a. The R-PC-TENG can be fixed in mountainous areas and cities to harvest wind and water flow energy for powering lights or some sensors, to realize self-powered environmental monitoring and wireless remote alarming. The application of the R-PC-TENG with diameter of 180 mm is demonstrated to have powered various electronic devices and energy storage units. Without a rectifying circuit, 944 green LEDs with a diameter of 5 mm connected in series are efficiently illuminated by the R-PC-TENG at the rotational speed of 60 rpm (Fig. 4b and Video S1). Subsequently, the R-PC-TENG application for the wind and water flow energy collecting are demonstrated in Video S2. As shown in Fig. 4c, the R-PC-TENG as a power source to drive electronics with a power management circuit (PMC). Various commercial capacitors can be charged quickly by the R-PC-TENG with the power management circuit (Fig. 4d), such as a 470 μF capacitor charged to 4 V within 5 s. In Fig. 4e, g and Video S3, the R-PC-TENG is utilized to power four parallel-connected commercial thermo-hygrometers, the fluctuation of voltage–time curve during operation shows that it works stably. It proves that the R-PC-TENG can provide power continuously for a long time in practical applications. Last, a commercial wireless transmission module is powered by the R-PC-TENG with a 2,000 μF capacitor, and wireless remote alarming can be realized when pressing the wireless transmission module (Fig. 4f and Video S4). The above applications strongly reveal the high output performance of the R-PC-TENG device in wind and water flow energy collecting and the applications for self-powered system in the field of intelligent environmental monitoring and wireless remote alarming.

## Conclusions

In summary, a DC output TENG device is achieved to harvest the wind energy based on the coupling of triboelectrification and electrostatic induction, which is inspired by the primary cell structure and its DC output characteristics. In addition, different working modes of PC-TENG can be designed according to different application scenarios, such as contact-separation mode CS-PC-TENG, free-standing mode FS-PC-TENG, and radially arrayed rotary mode R-PC-TENG. The PC-TENG device displays an excellent durability (maintaining 99%) after 100,000 cycles, owing to little wear on the surface of material through the ingenious structure design. Because of the high durability and large DC output performance of the R-PC-TENG, kinds of commercial capacitors are charged quickly and efficiently. Furthermore, the R-PC-TENG is applied to harvest wind energy and to illuminate 944 green LEDs with a diameter of 5 mm connected in series without a rectifying circuit. Finally, the R-PC-TENG is applied to power a commercial the wireless transmission module and four parallel-connected commercial thermo-hygrometers. This work offers an ideal strategy to harvest environment mechanical energy for Internet of Things via the R-PC-TENG with high output performance as power supplier for environmental monitoring and wireless remote alarming.

## Supplementary Information

Below is the link to the electronic supplementary material.

Supplementary file1 (PDF 289 kb)

Supplementary file2 (ZIP 52257 kb)
