# Supplementary material for: High Output Performance and Ultra-Durable DC Output for Triboelectric Nanogenerator Inspired by Primary Cell
Source: Nanomicro Lett. 2022 Aug 2;14:155. doi: 10.1007/s40820-022-00898-2 (PMC9346042; doi:10.1007/s40820-022-00898-2)
Supplement: Supplementary file 1 — Supplementary file1 (PDF 289 kb) [file 40820_2022_898_MOESM1_ESM.pdf]

Nano-Micro Letters

Supporting Information for

High Output Performance and Ultra-Durable DC Output for Triboelectric Nanogenerator Inspired by Primary Cell

Shaoke Fu[1], Wencong He[1], Huiyuan Wu[1], Chuncai Shan[1], Yan Du[1], Gui Li[1], Ping Wang[1], Hengyu Guo[1], Jie Chen[2,] *, Chenguo Hu[1,] *

[1]Department of Applied Physics, State Key Laboratory of Power Transmission Equipment & System Security and New Technology, Chongqing University, Chongqing 400044, P. R. China

[2] College of Physics and Electronic Engineering, Chongqing Normal University, Chongqing 401331, P. R. China

* Corresponding authors. E-mail: chenjie@cqnu.edu.cn (J. C.), hucg@cqu.edu.cn (C. Hu)

Supplementary Figures

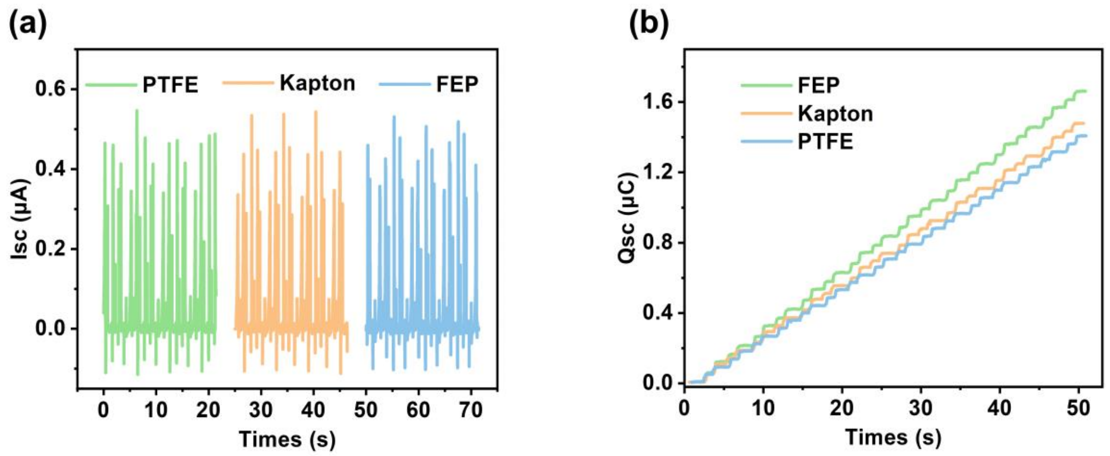

Fig. S1 The short-circuit current (**a**) and transferred charge (**b**) of the FS-PC-TENG with different electronegative materials

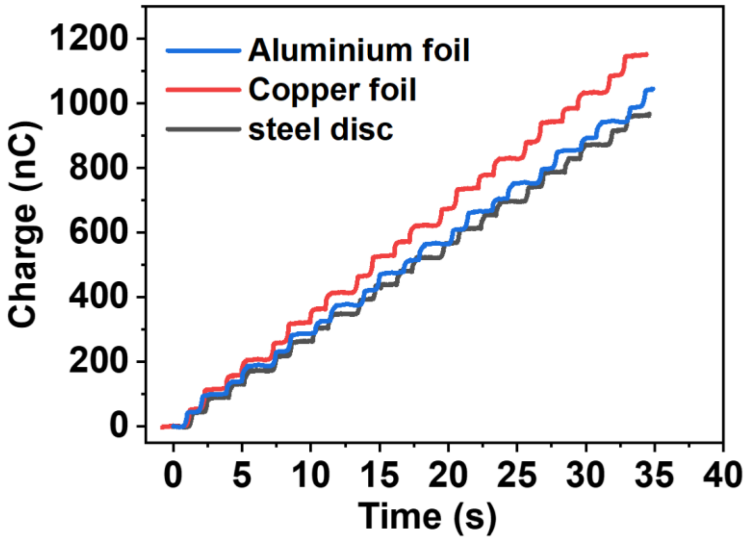

Fig. S2 The transferred charge of the FS-PC-TENG with different slider materials

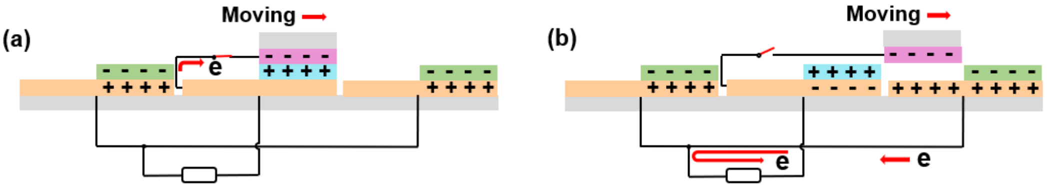

Fig. S3 The detailed working principle of the FS-PC-TENG. (**a**) The working principle of the FS-PC-TENG in stage iii. (**b**) The working principle of the FS-PC-TENG in stage iv

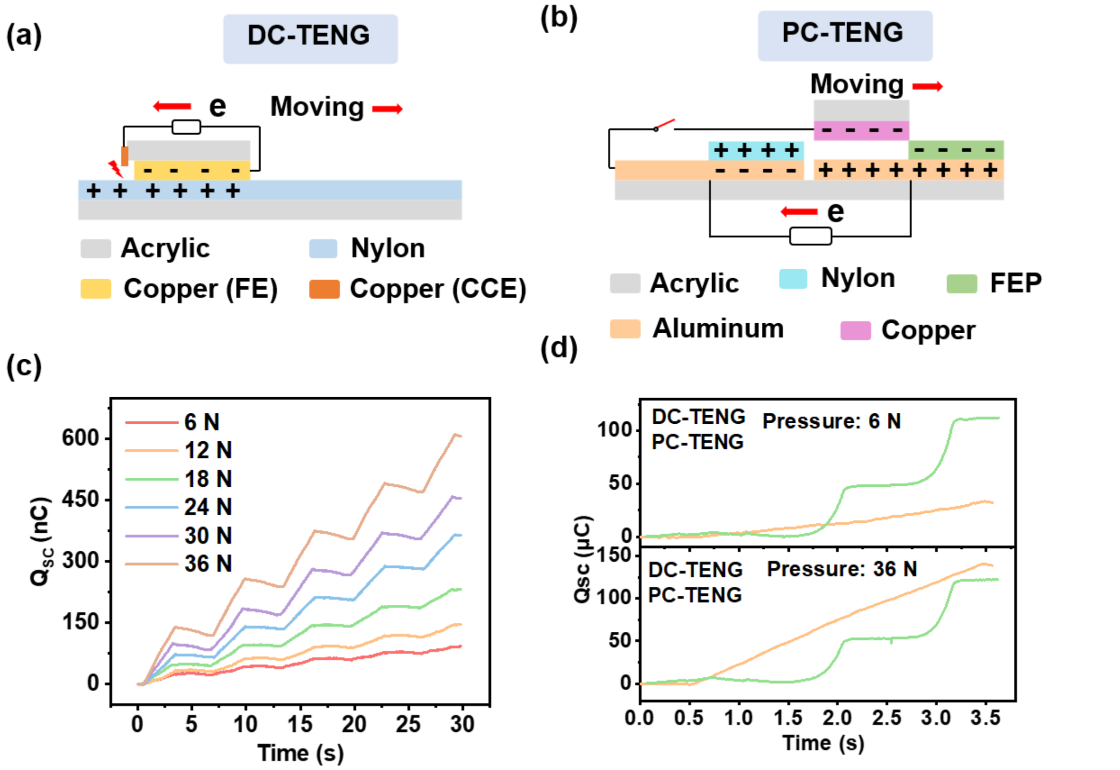

Fig. S4 The comparison of the output charge of PC-TENG with that of DC-TENG at different pressure forces. (**a**) Schematic diagram of DC-TENG based on air breakdown. (**b**) Schematic diagram of the PC-TENG based on contact electrification and electrostatic induction (**c**) the output charge of normal DC-TENG at different pressure forces. (**d**) The comparisons of the output charge of PC-TENG with that of normal DC-TENG at pressure forces of 6 N and 36 N, respectively

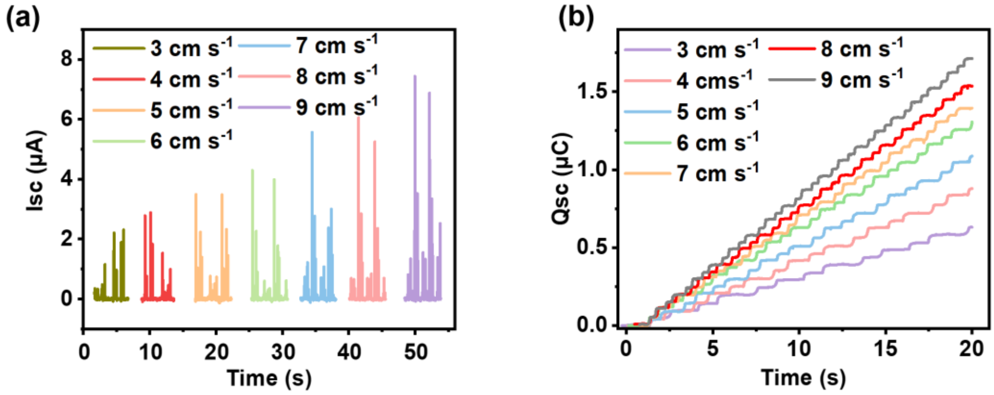

Fig. S5 The short-circuit current (**a**) and transferred charge (**b**) of the FS-PC-TENG with different sliding speeds
